# Supplementary figures and images for: Clinical validation of an open-access SARS-COV-2 antigen detection lateral flow assay, compared to commercially available assays
Source: PLoS One. 2021 Aug 17;16(8):e0256352. doi: 10.1371/journal.pone.0256352 (PMC8370603; doi:10.1371/journal.pone.0256352)

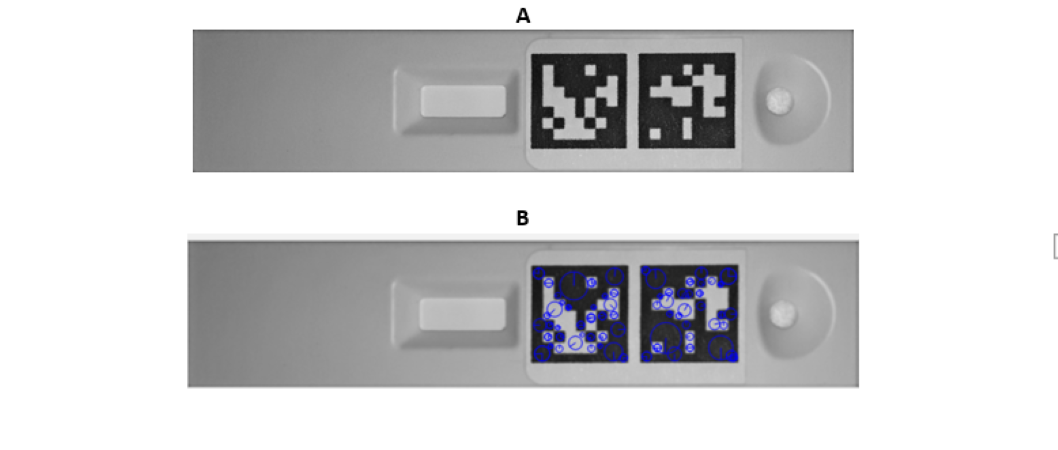

Supplement: S1 Fig — (A) Reference image of cassette used in this study, including the added sticker of ArUco codes. (B) Blue circles indicate the keypoints used in the SIFT algorithm for image recognition and transformation. Keypoints were filtered to ensure that pixels in the read window or sample pad were excluded from the matching process. (TIF) [file pone.0256352.s001.tif]

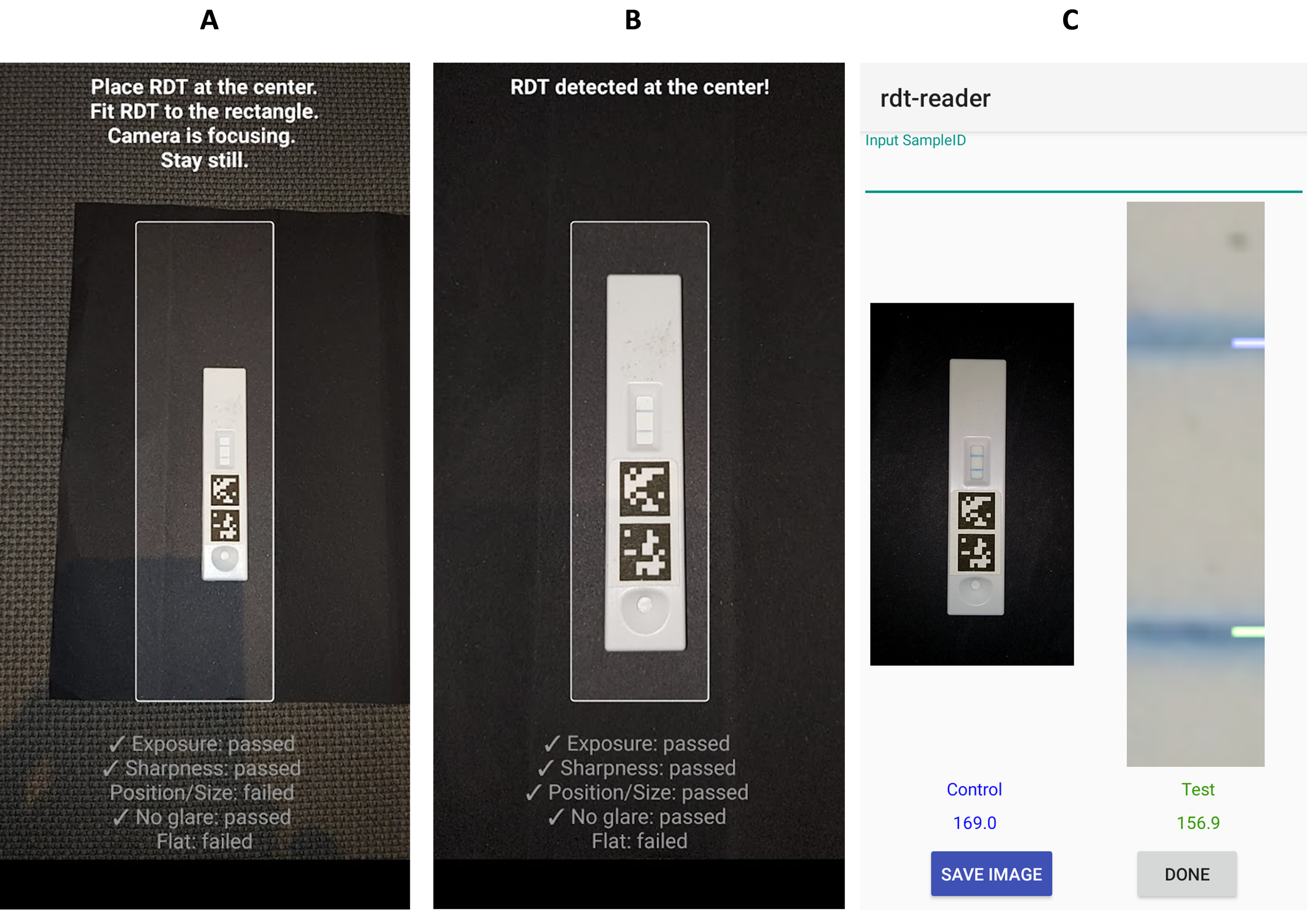

Supplement: S2 Fig — (A) Attempting to capture an image of the cassette—note the instructions to the user on top to move the phone and then keep it steady, as well as some but not all quality checks being passed. (B) Representative view of the cassette passing all quality checks, but just out of flatness range to facilitate screen grab. (C) Result window showing the full captured image, the extracted read window, and locations and intensities (peak height of red channel) of control and test lines, if found. (TIF) [file pone.0256352.s002.tif]

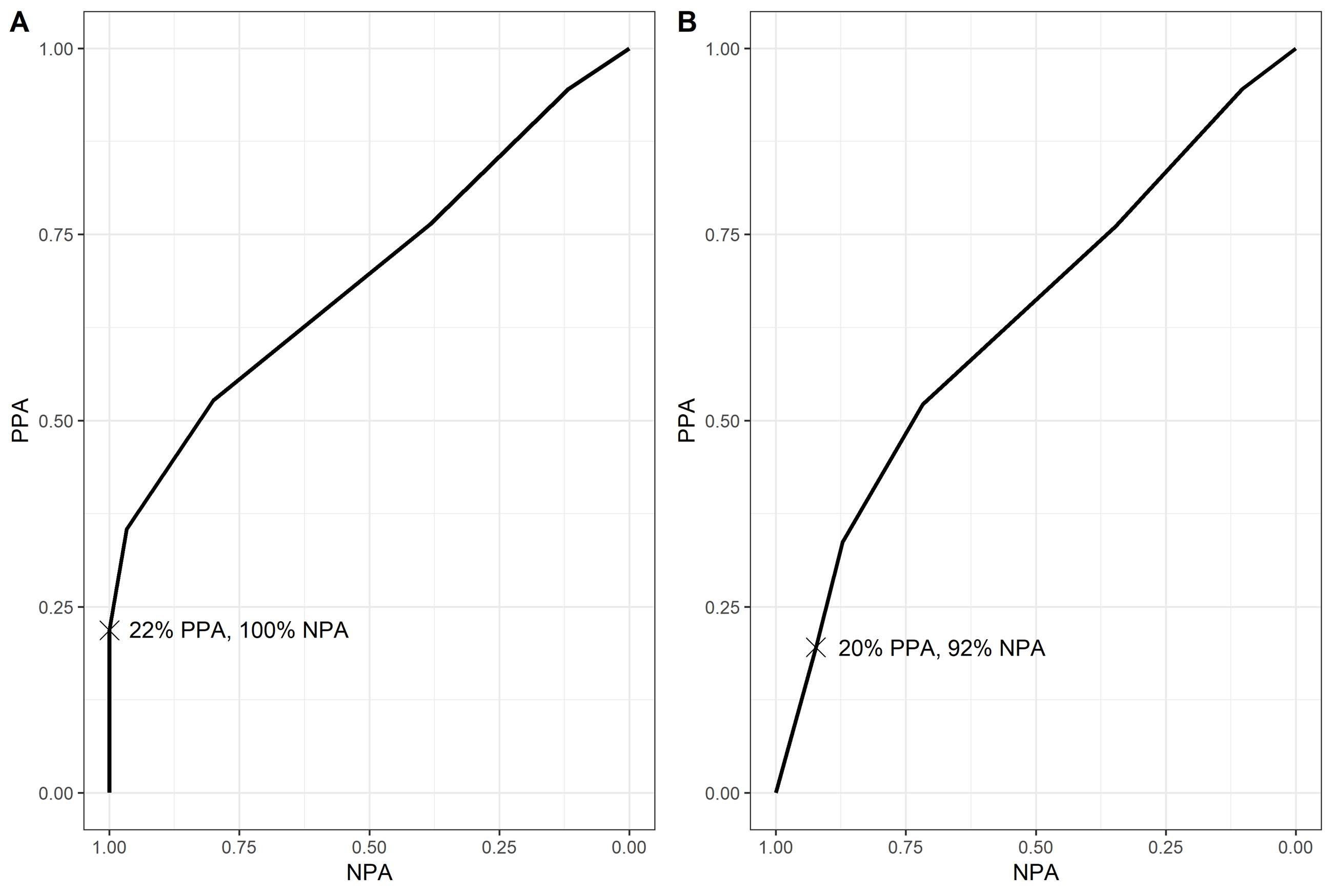

Supplement: S3 Fig — ROC curves for USmellIt relative to NP (A) and AN (B) PCR. A cut-off value of a USMELLIT score of 0 retains 100% specificity while providing 22% sensitivity for NP PCR while only 92% specificity is seen relative to AN PCR. The combination of an antigen-based test with a score of 0 offers potential improvement over an antigen test alone, as discussed in the main text. (TIF) [file pone.0256352.s003.tif]
