# Supplementary material for: Clinical validation of an open-access SARS-COV-2 antigen detection lateral flow assay, compared to commercially available assays
Source: PLoS One. 2021 Aug 17;16(8):e0256352. doi: 10.1371/journal.pone.0256352 (PMC8370603; doi:10.1371/journal.pone.0256352)
Supplement: S1 Table — Additionally, results from the McNemar mid-P test are shown for the sensitivity for cases with viral loads greater than and less than 1000 copies/μL. (DOCX) [file pone.0256352.s004.docx]

| **Assay** | **NP Swab PCR** | | **AN Swab PCR** | | | **NP Swab PCR Stratified Sensitivity** | | | **AN Swab PCR**  **Stratified Sensitivity** | | |
| --- | --- | --- | --- | --- | --- | --- | --- | --- | --- | --- | --- |
|  | **p value (Sensitivity)** | **p value (Specificity)** | | **p value (Sensitivity)** | **p value (Specificity)** | | **p value**  **< 1000 copies/µL** | **p value**  **> 1000 copies/µL** | | **p value**  **< 1000 copies/µL** | **p value**  **> 1000 copies/µL** |
| **BinaxNOW™ vs OA-LFA** | 0.0001 | 0.6250 | | 0.0039 | 0.2891 | | 0.0010 | 0.1250 | | 0.0040 | 1.0000 |
| **Sofia® vs OA-LFA** | 0.1460 | 0.6250 | | 0.3438 | 0.6875 | | 0.1250 | 0.6875 | | 0.3438 | 1.0000 |
| **BinaxNOW™ vs Sofia®** | 0.0117 | 0.5000 | | 0.0313 | 0.4531 | | 0.0391 | 0.25 | | 0.0313 | 1.0000 |

S1 Table. Results from McNemar’s mid-P value test comparing the sensitivity and specificity for all three tests against AN and NP swab PCR. Additionally, results from the McNemar mid-P test are shown for the sensitivity for cases with viral loads greater than and less than 1000 copies/µL.
