# Supplementary material for: Clinical validation of an open-access SARS-COV-2 antigen detection lateral flow assay, compared to commercially available assays
Source: PLoS One. 2021 Aug 17;16(8):e0256352. doi: 10.1371/journal.pone.0256352 (PMC8370603; doi:10.1371/journal.pone.0256352)
Supplement: S2 Table — (DOCX) [file pone.0256352.s005.docx]

| **Assay** | **NP Swab PCR** | | **AN Swab PCR** | |
| --- | --- | --- | --- | --- |
|  | **Sensitivity (95% CI)** | **Specificity (95% CI)** | **Sensitivity (95% CI)** | **Specificity (95% CI)** |
| **OA-LFA** | 69% (60%-78%), 75/108​ | 97% (88%-100%), 57/59​ | 83% (74%-90%) 75/90​ | 97% (91%-100%), 75/77​ |
| **OA-LFA or USmellIt Score = 0**​ | 75% (66%-83%), 81/108​ | 97% (88-100%) 57/59​ | 83% (74%-90%) 75/90​​ | 90% (81%-95%), 69/77​ |
| **Sofia®** | 74% (64%-82%), 81/110​ | 98% (91%-100%), 59/60​ | 86% (77%-92%), 79/92​ | 96% (89%-99%), 75/78​ |
| **Sofia® or USmellIt Score = 0** | 80% (71%-87%), 88/110​ | 98% (91%-100%), 59/60​​ | 88% (80%-94%) 81/92​ | 90% (81%-95%), 70/78​ |
| **BinaxNOW™** | 82% (73%-88%), 89/109​ | 100% (94%-100%), 60/60​ | 91% (84%-96%), 84/92​ | 94% (85%-98%), 72/77​ |
| **BinaxNOW™ or USmellIt Score = 0** | 84% (76%-91%), 92/109​ | 100% (94-100%), 60/60​ | 91% (84%-96%) 84/92​ | 90% (81%-95%), 69/77​​ |

S2 Table. Sensitivity and specificity of each test relative to AN and NP PCR with or without the inclusion of the zero USMELLIT criterion.
