# Supplementary material for: Clinical validation of an open-access SARS-COV-2 antigen detection lateral flow assay, compared to commercially available assays
Source: PLoS One. 2021 Aug 17;16(8):e0256352. doi: 10.1371/journal.pone.0256352 (PMC8370603; doi:10.1371/journal.pone.0256352)
Supplement: S1 File — (DOCX) [file pone.0256352.s007.docx]

**S1 File**

**1. Mobile reader application modifications and additions.** The following provides further details on development and use of the mobile app reader for LFA image interpretation.

**1.1 Cassette Detection**

As in the original app by Park et al. (12), the cassette is identified in the captured image using the Scale Invariant Feature Transform (SIFT) feature detection algorithm based on a reference image of the cassette (Fig S1A). Since the cassettes used in this study were otherwise blank, a sticker containing two ArUco codes was applied to each cassette to identify the cassette in the image and to determine its orientation. The size and scale of the ArUco markers provided a large number of high-contrast keypoints (Fig S1B) that are less susceptible to lighting conditions than less structured markings or higher resolution 2D codes such as QR codes. For this work, the reference image was post-processed to remove the keypoints that were associated with glare or shadowing when capturing the reference image. Once this processed reference image was in place, keypoint matching and subsequent transforms were performed as detailed in Park et al. (12).

**1.2 Phone level check.** As seen in Fig S2, the app runs a number of real-time quality checks on the current video frame. Here, an additional check was added to determine whether the phone was level to the ground (“flat”). This check was added to prevent the acquisition of overly skewed images, as well as to prevent shadows being cast onto the paper read window from the edges of the trough. To implement this feature, the standard Android API was used to obtain tilt information from the phone’s accelerometer. If the tilt in any direction was greater than five degrees, the quality check did not pass.

**1.3 High resolution still image capture.** To obtain potentially higher quality images, a function was added to capture a high resolution still image immediately after the video image passed the application’s quality checks. The Android camera settings were customized for each image stream, where the camera algorithms for video prioritize frame rate for a good user experience when framing the scene, and the camera settings for the still image capture prioritize image quality. It was found that the quality of the high-resolution image acquired using the native camera quality checks for a still image produced reliable quality without the need for a secondary quality check performed by the application. The high-resolution image was saved locally and then transformed and further processed using Python scripts with the same algorithms implemented in the application for the video images.

**1.4 Image and data processing updates.** As in the original app framework, once an image was successfully acquired, the region of interest (i.e. read window) was cropped and processed. The general steps involved in processing captured images include (1) read in preset configurations about the cassette, including annotations of lines, line positions, and offset, etc.; (2) perform perspective correction on the raw image; (3) locate the test strip based on default locations from reference image; (4) convert image to grayscale for quality checks; and (5) convert image color space to RGB and select red image channel for 1D signal calculation.

The 1D signal was calculated by averaging a cropped test strip image across its width using the selected channel. Further processing steps and peak detection methods were then altered from the original app framework. In particular, the calculated 1D signal was smoothed using a 2^nd^ order Savitzky-Golay filter, and then a parameterized peak detection algorithm was applied to the smoothed 1D signal. The peak detector put two constraints on reported peaks. To be identified as a peak, the peak must be a local maximum within a preset minimum distance. Thus, only the most prominent peak could be picked up during the search. The prominence, or peak height above baseline, also needed to be greater than a threshold value to be reported. To calculate this peak height above baseline, a linear baseline was fitted across the identified peak’s calculated peak width. Then, a peak to baseline distance was calculated and reported as peak height.
