# Supplementary material for: Clinical validation of an open-access SARS-COV-2 antigen detection lateral flow assay, compared to commercially available assays
Source: PLoS One. 2021 Aug 17;16(8):e0256352. doi: 10.1371/journal.pone.0256352 (PMC8370603; doi:10.1371/journal.pone.0256352)
Supplement: Attachment — Submitted filename: Reviewer PlosOne PONE-D-21-19695 yb.docx [file pone.0256352.s008.docx]

Research article: PONE-D-21-19695

Title: Clinical validation of an open-access SARS-COV-2 antigen detection lateral flow assay, compared to commercially available assays

Bacham and colleagues have submitted an article presenting a clinical validation of an open-access lateral flow assay (OA-LFA) design using commercially available materials and reagents, along with RT-qPCR, BinaxNOW® and Sofia® rapid diagnostic tests. They found that the open-access LFA meets the minimum WHO target product profile for a rapid test with a positive predictive agreement with NP sampling of 69% (60% -78%) for OA-LFA as compared to 74% (64% - 82%) for Sofia®, and 82% (73% - 88%) BinaxNOW™ in adults patients with COVID-19 symptoms less than 7 days. Bacham *et al* include the evaluation of a reader app that could largely mirror visual interpretation of Labs

Test.

There are however few some points to address before being considered for publication in PlosOne

1. The authors have rightly chosen to calculate the positive and negative predictive agreement to assess the performance of the OA-LFA. However, most of the evaluation of diagnostic tests use Sensitivity, specificity, PPV and NPV. We recommend the authors to include those parameters using the RT-qPCR as the reference as done in most rapid diagnostic tests evaluation. This will make easier the comparison with other evaluations. Moreover in line 233-235 the authors refer to the WHO TPP requirement of 80% sensitivity and 97 specificity.
2. In table 1 the authors provide important data to appreciate the performance of each test. It would be better to also provide the 2x2 table for each vs RT-qPCR results. If this is not possible in the main text they could be added in the supplementary
